# New Insights into the Mechanism of Nucleation of ZrO_2_ Inclusions at High Temperature

**DOI:** 10.3390/ma15227960

**Published:** 2022-11-10

**Authors:** Yutang Li, Linzhu Wang, Chaoyi Chen, Shufeng Yang, Xiang Li

**Affiliations:** 1 School of Materials and Metallurgy, Guizhou University, Guiyang 550025, China; 2 State Key Laboratory of Public Big Data, Guizhou University, Guiyang 550025, China; 3 School of Metallurgical and Ecological Engineering, University of Science and Technology Beijing, Beijing 100083, China; 4 Department of Materials and Metallurgy Engineering, Guizhou Institute of Technology, Guiyang 550003, China

**Keywords:** inclusion, ZrO_2_, nucleation mechanism, first principles

## Abstract

It is difficult to observe the nucleation mechanism of inclusions in real-time. In this study, the nucleation process of zirconium oxide inclusions was systematically studied by classical nucleation theory and first principles. Zr deoxidized steel with 100 ppm Zr addition was processed into metallographic samples for scanning electron microscopy energy-dispersive spectroscopy observation. The electrolytic sample was analyzed by micro X-ray diffraction and transmission electron microscopy, and the zirconium oxide in the sample was determined to be ZrO_2_. The nucleation rate and radius of the ZrO_2_ inclusions were calculated by classical nucleation theory, and they were compared with the experimental values. There was a considerable difference between the experimental and theoretical values of the nucleation rate. The effect of the nucleation size was analyzed by first-principles calculation, and the thermodynamic properties of ZrO_2_ clusters and nanoparticles were analyzed by constructing (ZrO_2_)*_n_* (*n* = 1–6) clusters. The thermodynamic properties of ZrO_2_ calculated by first principles were consistent with the values in the literature. Based on two-step nucleation theory, the nucleation pathway of ZrO_2_ is as follows: Zr_atom_ + O_atom_ → (ZrO_2_)*_n_* → (ZrO_2_)_2_ → core (ZrO_2_ particle)–shell ((ZrO_2_)_2_ cluster) nanoparticle → (ZrO_2_)_bulk_.

## 1. Introduction

The mechanical properties and service life of steel are seriously affected by inclusions. After the proposal of the concept of oxide metallurgy [1], researchers have realized that it is important to control the size of inclusions [2,3,4] rather than increase the smelting cost [5,6,7,8]. Formation of inclusions begins with nucleation, and exploring the nucleation mechanism and properties of inclusions in steel is important to control the inclusion size. However, owing to the high speed of inclusion nucleation and high smelting temperature, the inclusion-nucleation process is difficult to detect and observe directly. Therefore, research on the inclusion of nucleation in steel is a challenge.

Researchers have found that Zr-containing inclusions can promote acicular ferrite transformation in Zr-deoxidized steel under certain conditions [9,10,11,12,13]. Zr-containing inclusions in Zr-deoxidized steel also play an important role in oxide metallurgy. On the one hand, they can induce nucleation of intragranular ferrite. There are several theories about the nucleation mechanism of ferrite. The change in the chemical composition of austenite around inclusions promotes nucleation. Inclusions and precipitates are coherent with ferrite to reduce the potential nucleation barrier and promote nucleation. In addition, the strain energy caused by the difference in thermal shrinkage between inclusions and austenite, as well as inclusions acting as an inert interface, promotes nucleation [14]. Among the proposed theories, the Mn-depleted-zone mechanism formed by the precipitation of MnS on ZrO_2_ is considered to be one of the most effective mechanisms for intragranular ferrite nucleation [15,16]. On the other hand, Zr-containing inclusions play an important role in controlling the distribution of MnS inclusions in the steel. The density of ZrO_2_ is close to that of liquid steel, and the volume is small. Therefore, it is not easy for ZrO_2_ to float up in liquid steel, and it is easier for ZrO_2_ to disperse and distribute in steel to improve the distribution of sulfide through heterogeneous nucleation. Theoretical calculation shows that the lattice mismatch degree between MnS and ZrO_2_ is only 5.2%, and ZrO_2_ is the most effective nucleation core to promote MnS nucleation [17]. The thermodynamics from first-principles calculation show that Mn will diffuse into ZrO_2_ because ZrO_2_ has cationic vacancies and can absorb Mn [18]. Whether it induces intragranular ferrite nucleation or acts as a heterogeneous nucleation core, the key is the size control of ZrO_2_. However, there has been limited research on the nucleation mechanism and size control of ZrO_2_. In addition, the inclusion-nucleation speed is fast and the smelting temperature is high, so the inclusion-nucleation process is difficult to detect and observe directly.

To study the evolution of alumina inclusions at the atom scale, Wang et al. [19,20,21] investigated the cluster structure from experimental and theoretical aspects, and they proposed a two-step nucleation mechanism. Using quenching and three-dimensional atomic-probe detection technology, Zhao et al. [22] captured the intermediate structure of titanium oxide, and they proposed the cluster-assisted nucleation mechanism. Yang et al. [23] simulated the growth process of clusters by molecular dynamics, and they found that clusters grow through collision. However, there has been limited research on the nucleation of zirconium oxide. Thus, it is necessary to study the nucleation process of zirconium oxide.

In this study, a high-temperature Zr deoxidation experiment was carried out, and the characteristics of the zirconium oxide inclusions in liquid steel, such as the composition, morphology, size, quantity, and area density, were statistically analyzed. According to classical thermodynamic nucleation theory, the relationships between the solute element activities and the nucleation radius and nucleation rate of inclusions were obtained, and the theoretical nucleation rate was compared with the experimental nucleation rate. Cluster models of zirconium oxide were constructed by Materials Studio software. The cluster structure and thermodynamic properties of nanoparticles after geometric optimization were calculated, and the accuracy of the first-principles calculation was verified. By combining the high-temperature experimental results, classical nucleation calculations, and first-principles analysis, the nucleation mechanism of zirconium oxide inclusions is proposed.

## 2. Materials and Methods

### 2.1. Sample Preparation

Pure iron was used as the raw material, and it was heated in a Si–Mo heating electric resistance furnace (Braveman Special Testing Furnace CO. LTD., Luoyang, Henan, China). The chemical composition of the pure iron sample is shown in Table 1. The pure iron sample was heated to 1873 K (1600 °C) in an alumina crucible in the Si–Mo heating electric resistance furnace. After the temperature was maintained at 1873 K for 30 min, the Zr–Fe alloy (60% Zr) wrapped in a high-purity iron belt was added to the melted pure iron, followed by stirring for 10 s to ensure uniform distribution of the Zr–Fe alloy. Finally, 120 s after adding the Zr–Fe alloy, samples were removed in quartz tubes, followed by quenching in water. The whole experimental process was protected by high-purity argon gas.

The inclusions were extracted by electrolysis. A copper plate was used as the cathode, and the sample was the anode. After electrolysis, the anode was placed in anhydrous ethanol. By ultrasonic cleaning, the inclusions attached to the anode were dispersed in anhydrous ethanol. Finally, the inclusions extracted by electrolysis were analyzed by micro X-ray diffraction (μXRD, Bruker D8 Advance, Bruker, Berlin, Germany) and transmission electron microscopy (TEM, JEOL JEM-F200, JEOL, Tokyo, Japan).

### 2.2. Microstructure and Composition Characterization

To measure the composition and morphologies, the samples were processed into ⌀5 mm × 15 mm metallographic samples. Scanning electron microscopy with energy-dispersive X-ray spectroscopy (SEM-EDS) (EM-30PLUS, COXEM, Daejeon, Korea) was then performed. In addition, the samples were processed into ⌀5 mm × 10 mm bars for total oxygen and nitrogen content detection by the fusion-infrared absorption method. The oxygen and nitrogen contents were measured three times to investigate the uniformity of the total oxygen and nitrogen contents in the molten iron. In addition, the total Zr content was measured by inductively coupled plasma–atomic emission spectroscopy. The chemical contents of oxygen, nitrogen, and zirconium are given in Table 2.

To measure the composition of the inclusions in the steel after zirconium addition, the inclusions extracted by electrolysis were observed by μXRD and TEM. The results provided an experimental reference for subsequent first-principles calculation of inclusion crystal-type selection.

### 2.3. Nucleation Calculation

According to classical nucleation theory, the critical nucleation size and nucleation rate *I* [cm^−3^·s^−1^] can be calculated by [24]

(1) $$\ln I=\frac{16\pi \gamma_{SL}^{3}V_{O}^{2}}{3k_{B}R^{2}T^{3}}\left(\frac{1}{{\left(\ln S_{O}^{*}\right)}^{2}}-\frac{1}{{\left(\ln S_{O}\right)}^{2}}\right)$$

(2) $$r_{C}=-\frac{2\gamma_{SL}}{∆G_{V}}=\frac{2r_{SL}V_{O}}{RT\ln S_{O}}$$

where *k_B_* is the Boltzmann constant (1.38 × 10^−23^ J/K), *R* is the gas constant (8.314 J/(mol K)), *T* [K] is the absolute temperature, and *V_O_* [m^3^/mol] is the molar volume of oxide. $\gamma_{SL}$ [J/m^2^] is the interfacial energy between the oxide and liquid steel, and it can be expressed by Young’s equation:

(3) $$\gamma_{SL}=\gamma_{SV}-\gamma_{LV}\cos\theta$$

(4) $$\gamma_{LV}=1.75-0.279\ln\left(1+140\cdot a_{O}\right)$$

where $\gamma_{SV}$ is the surface energy of the solid inclusion, $\gamma_{LV}$ is the surface energy of the liquid steel, which has been calculated in previous studies [25,26], and θ is the contact angle between liquid steel and the inclusion, as illustrated at Table 3.

The experimental values of the nucleation rate *I* can be obtained by [24]

(5) $$I=\frac{f_{v}}{\frac{4}{3}\pi r^{3}\cdot t}$$

where *t* is the nucleation time (generally taken to be 0.2 s [29]) and *r* is the critical nucleation radius obtained by Equation (2). *f_v_* is the volume fraction of oxide particles: [24]

(6) $$f_{v}=\frac{\rho_{Fe}}{\rho_{ZrO_{2}}}\cdot \frac{M_{ZrO_{2}}}{xM_{Zr}}\cdot \left[\mathrm{ppm}\;\mathrm{insol}.\;\mathrm{Zr}\right]\times 10^{-6}$$

where *ρ_Fe_* and *ρ_ZrO2_* are the densities of Fe and ZrO_2_, respectively (*ρ_Fe_* = 7.8 g/cm^3^, *ρ_ZrO2_* = 5.85 g/cm^3^) [30], *M_ZrO2_* is the molecular weight of ZrO_2_, *M_zr_* is the atomic weight of Zr, and [ppm insol. Zr] represents the Zr content.

### 2.4. First-Principles Calculation

The DMol [3] module based on density functional theory in the Materials Studio software package (Materials Studio8.0, Accelrys, California, America) was used for cluster optimization and thermodynamic property calculation. The Broyden–Fletcher–Goldfarb–Shanno mechanism was used for geometric optimization. The Perdew–Burke–Ernzerhof functional with the generalized gradient approximation was selected as the electron exchange–correlation potential function [31].

## 3. Results

### 3.1. Inclusion Characterization

**Figure 1:**
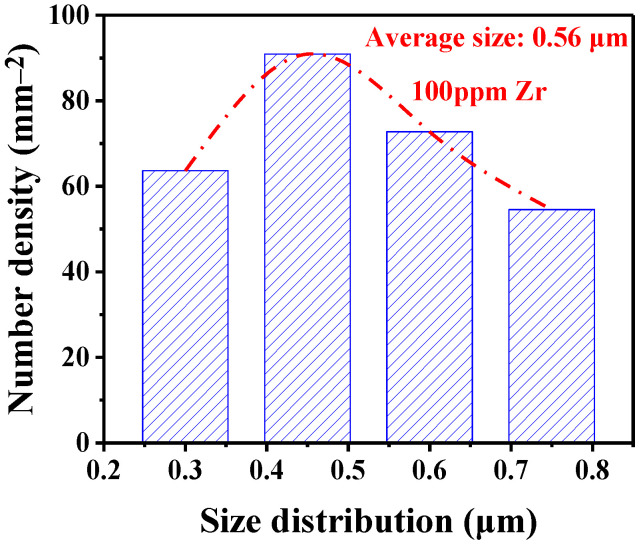
Size distribution of ZrO_2_ in Zr deoxidized steel.

To obtain the size distribution of the ZrO_2_ inclusions, 100 SEM images were continuously taken at 5000× magnification, and the total observed area was 0.11 mm^2^. The average size of the ZrO_2_ inclusions in the sample was 0.56 μm, and the size distribution of ZrO_2_ inclusions approximately approached a normal distribution (Figure 1).

**Figure 2:**
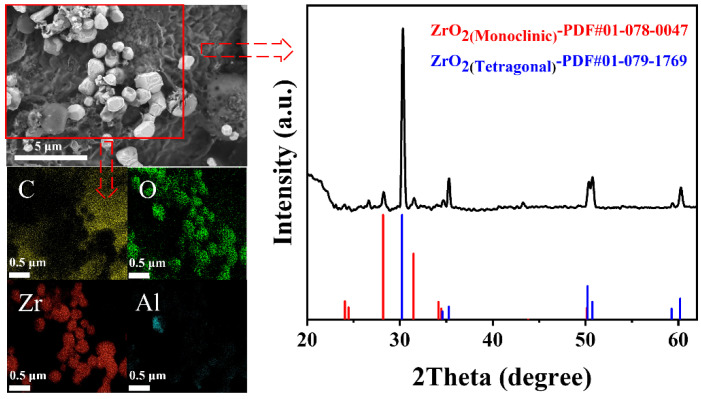
Morphology, composition, and X-ray diffraction pattern of the inclusions in Zr deoxidized steel.

The inclusions extracted by electrolysis were characterized by µXRD, and the morphology and composition were analyzed by SEM-EDS (Figure 2). From the mapping image, the inclusions in the Zr deoxidized steel were zirconium oxide. A few Al inclusions were also detected because of the trace amount of Al in the raw materials. The results of µXRD suggested that the zirconium oxide was ZrO_2_. In addition, both monoclinic and tetragonal ZrO_2_ were detected. This may be because of the transformation of tetragonal ZrO_2_ to monoclinic ZrO_2_ during rapid cooling.

### 3.2. Classical Nucleation Calculation

**Figure 3:**
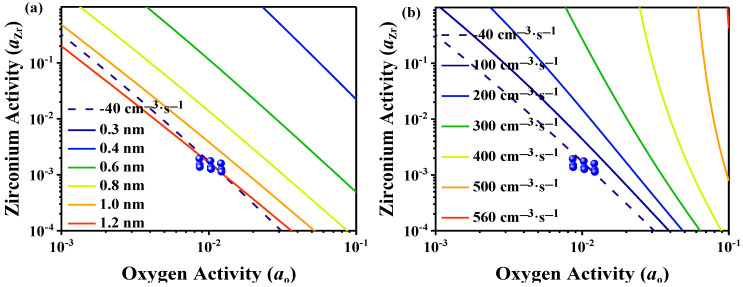
Relationships between the activities of the solute elements and the (**a**) critical nucleation radius and (**b**) nucleation rate of ZrO_2_.

According to classical nucleation theory, the relationship between the critical nucleation radius of ZrO_2_ and the activities of the solute elements at 1873 K is shown in Figure 3a. When the Zr activity is in the range 0.0001–1, and the oxygen activity is in the range 0.0001–0.1, the critical nucleation radius of ZrO_2_ is 0.3–1.2 nm. The relationship between the nucleation rate and solute element activities is shown in Figure 3b. When the Zr activity is in the range 0.0001–1, and the oxygen activity is in the range 0.001–0.1, the critical nucleation rate of ZrO_2_ is in the range 100–560 cm^−3^·s^−1^. The points in Figure 3 are the experimentally measured Zr and oxygen activities.

To obtain the activities of zirconium and oxygen, the composition of the zirconium deoxidized steel and corresponding thermodynamic data were substituted into

(7) $$\;a_{i}=f_{i}\left[\mathrm{mass}\%\;i\right]$$

(8) $$\log f_{i}=\sum e_{i}^{j}\left[\mathrm{mass}\%\;i\right]$$

where $a_{i}$, $f_{i}$, and $\left[\mathrm{mass}\%\;i\right]$ are the activity, activity coefficient, and concentration of element *i*, respectively, and $e_{i}^{j}$ is the first-order interaction coefficient (Table 4).

The experimentally estimated value of ln *I* can be calculated by Equations (5) and (6). The experimental value of ln *I* was 57 cm^−3^·s^−1^. From Figure 3, the theoretical value of ln *I* is approximately −40 cm^−3^·s^−1^. Therefore, the experimental value of the nucleation rate *I* was approximately 40 orders of magnitude larger than the theoretical value.

### 3.3. First-Principles Calculations

According to the two-step nucleation mechanism, the nucleation process of inclusions in liquid steel should include the multiphase deoxidation reaction, which can be expressed by the following two steps [32,33,34,35]. In the first step, the deoxidized elements in the molten steel melt and dissolve, and the deoxidized element atoms react with the dissolved oxygen in the molten steel to form oxide clusters. In the second step, the clusters combine to form cluster aggregates. The cluster aggregates then form critical crystal nuclei.

#### 3.3.1. Structures of (ZrO_2_)*_n_* Clusters

With increasing *n* value, the average bond length of the (ZrO_2_)*_n_* cluster initially slightly increases, and it finally fluctuates at approximately 2.0 Å (Table 5). The nucleon binding ability in the nucleus is stronger and more stable for larger average binding energy (*E_bin_*). The average binding energies of the (ZrO_2_)*_n_* (*n* = 1–6) clusters are negative (Table 5), indicating that the binding between nuclei is relatively stable.

With an increasing number of atoms (*n* = 1–6), the average binding energy of the (ZrO_2_)*_n_* cluster decreases, especially between the (ZrO_2_)_1_ and (ZrO_2_)_2_ clusters. This may be because of the energy error caused by the different numbers of atoms in different clusters. Therefore, the energy gap was used to compare the stabilities of the clusters. The energy gap is the difference between the lowest unoccupied molecular orbital (LUMO) and the highest occupied molecular orbital (HOMO). The energy gap reflects the ability of electrons to transition from an occupied orbital to an empty orbital, and it represents the ability of molecules to participate in chemical reactions. The system is more stable for a larger energy gap [36].

**Figure 4:**
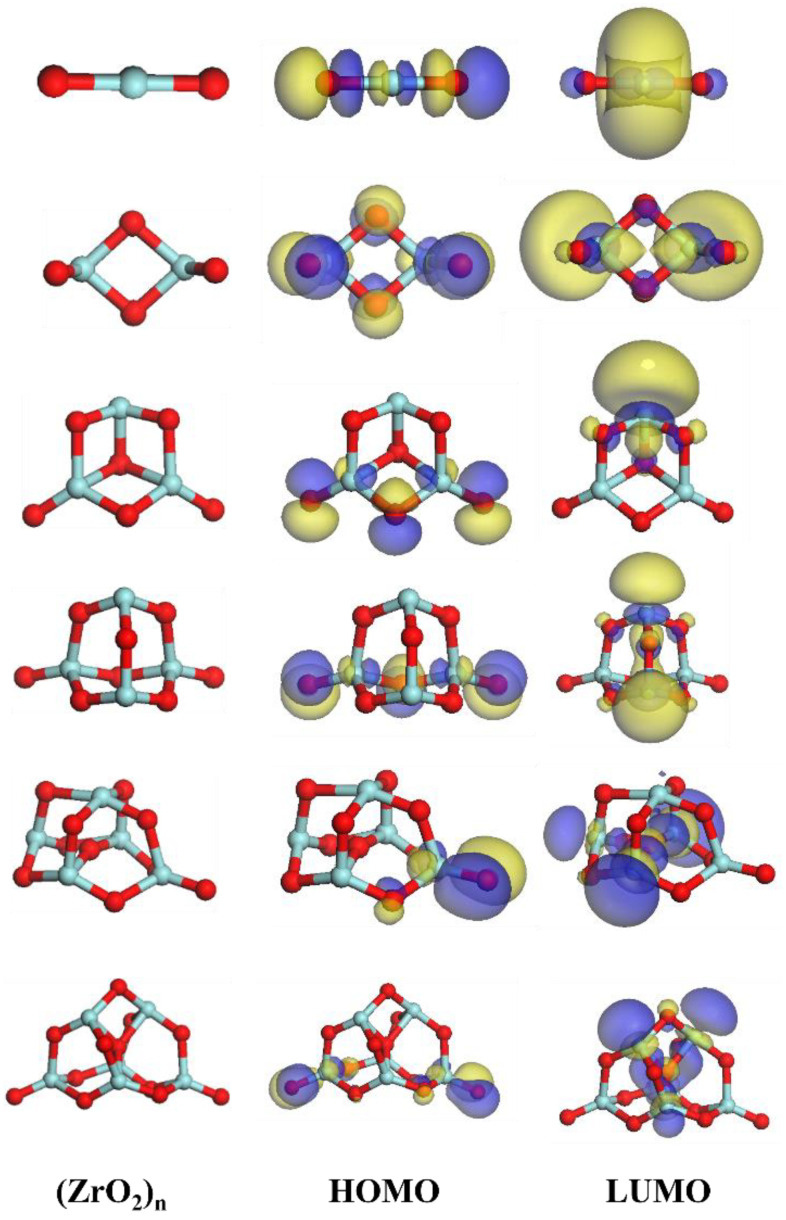
HOMOs and LUMOs of the (ZrO_2_)*_n_* clusters.

The energy gaps of the (ZrO_2_)*_n_* clusters are given in Table 6. The (ZrO_2_)_2_ cluster has the largest energy gap, indicating that the (ZrO_2_)_2_ cluster is the most stable of the (ZrO_2_)*_n_* clusters (*n* = 1–6). The LUMOs and HOMOs of the (ZrO_2_)*_n_* clusters are shown in Figure 4. The blue and yellow area denote the orbitals of electron cloud, where the color is to distinguish the plus or minus of orbital wave function.

#### 3.3.2. Thermodynamic Properties of the (ZrO_2_)*_n_* Clusters

**Figure 5:**
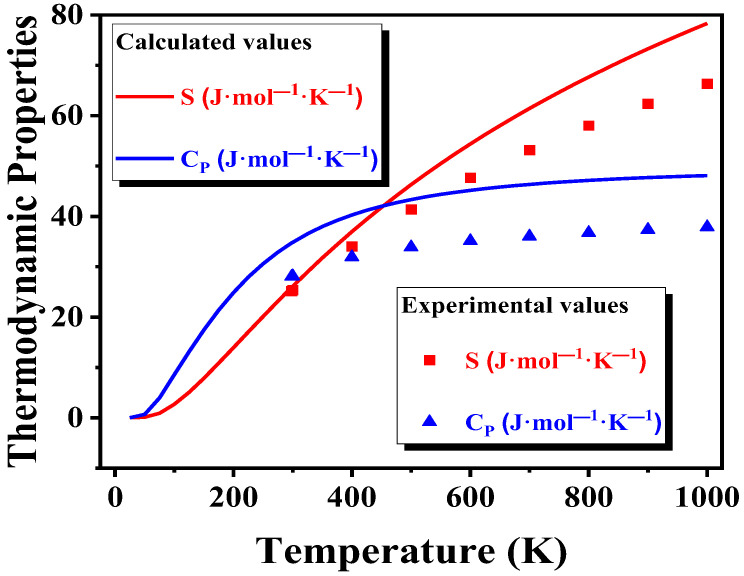
Thermodynamics properties of ZrO_2_.

The thermodynamic properties of ZrO_2_ are shown in Figure 5. Where *S* is the entropy, and *C_p_* is the heat capacity. In this study, the ZrO_2_ crystal structure was tetragonal. The lines and points represent the calculated thermodynamic properties and values in the literature, respectively [37]. In the temperature range of 0–1000 K, there is a certain degree of deviation between the calculated and the values in the literature, but the variation trend of the thermodynamic properties with temperature is consistent. To the best of our knowledge, the reason that caused deviation is mainly the machine error derived from the thermochemical software package, and it was not possible to meet strict consistency criteria when combining data from various sources to form a data set for a substance. Another reason is that phase transformation occurred between monoclinic and tetragonal ZrO_2_. In general, the calculated value is in good agreement with the value from the literature.

#### 3.3.3. Gibbs Free Energy Changes of (ZrO_2_)*_n_* Clusters and Nanoparticles

**Figure 6:**
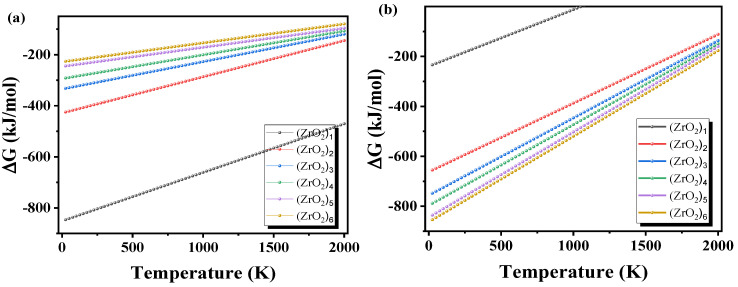
(**a**) Formation Gibbs free energy curves of (ZrO_2_)*_n_*. (**b**) Formation Gibbs free energy curve of ZrO_2(s)_.

The formation Gibbs free energy (Δ*G*) curves of the (ZrO_2_)*_n_* clusters and ZrO_2(s)_ are shown in Figure 6a,b, respectively. The formation Gibbs free energies of (ZrO_2_)*_n_* (*n* = 1–6) are negative, suggesting the (ZrO_2_)*_n_* (*n* = 1–6) clusters form. However, the formation Gibbs free energy change from (ZrO_2_)_1_ to ZrO_2(s)_ is positive, so the reaction from (ZrO_2_)_1_ to ZrO_2(s)_ does not occur when the temperature is greater than 1000 K.

**Figure 7:**
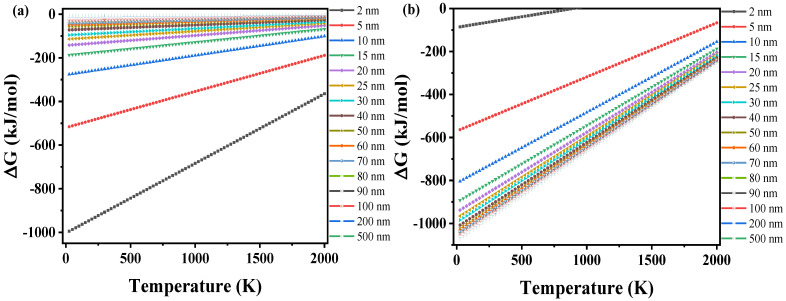
(**a**) Gibbs free energy change from nanoscale ZrO_2_ to ZrO_2(bulk)_. (**b**) Gibbs free energy changes from the Zr and O atoms to nanoscale ZrO_2_.

The Gibbs free energy changes from nanoscale ZrO_2_ to ZrO_2(bulk)_ and from the Zr and O atoms to nanoscale ZrO_2_ are shown in Figure 7a,b, respectively. The Gibbs free energy of the macroscale ZrO_2_ crystal is less than zero, indicating that nanoscale ZrO_2_ will spontaneously transform to the macroscale ZrO_2_ crystal.

## 4. Discussion

**Figure 8:**
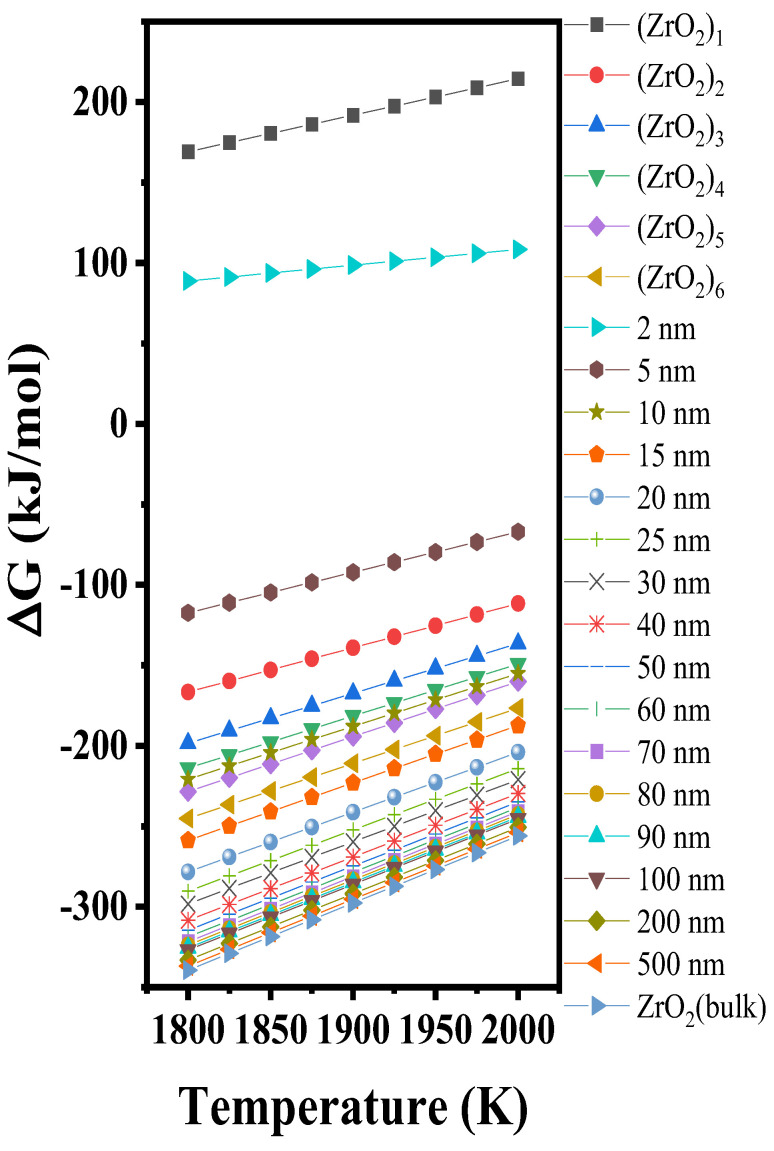
Formation Gibbs free energy changes of (ZrO_2_)*_n_* clusters and various ZrO_2_ nanoparticles.

The formation of Gibbs free energy changes of the (ZrO_2_)*_n_* clusters and various ZrO_2_ nanoparticles are shown in Figure 8. Most of the Gibbs free energy change curves of the (ZrO_2_)*_n_* clusters are higher than those of the ZrO_2_ nanoparticles, which is in agreement with the law of the step-by-step change of the thermodynamic stability., from the perspective of the gradual decrease of the energy, this law indicates that the formation process of ZrO_2_ occurs from the atoms to clusters to nanoscale crystal particles to the macroscale crystal.

**Figure 9:**
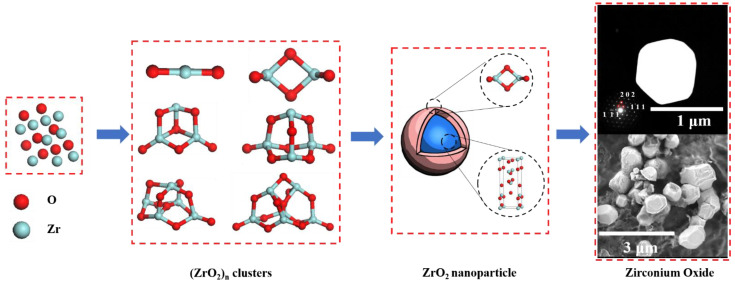
Nucleation process of zirconium oxide at 1873 K.

The surfaces of nanoparticles usually contain two or three atomic layers [38,39], and the average length of the Zr–O bond is approximately 0.214 nm. Therefore, the surface atomic layer of zirconium oxide nanoparticles is approximately 0.428–0.642 nm thick. From Table 4, the sizes of the (ZrO_2_)*_n_* clusters are also within this range. In addition, from Table 5, the energy gap of the (ZrO_2_)_2_ cluster is the largest, indicating that this cluster is the most stable. Therefore, the (ZrO_2_)_2_ cluster may be the atomic layer on the surface of the ZrO_2_ nanoparticle. Based on the two-step nucleation theory, it is speculated that the nucleation pathway of ZrO_2_ is Zr_atom_ + O_atom_ → (ZrO_2_)*_n_* → (ZrO_2_)_2_ → core (ZrO_2_ particle)–shell ((ZrO_2_)_2_ cluster) nanoparticle → (ZrO_2_)_bulk_. The nucleation process of ZrO_2_ at 1873 K is shown in Figure 9.

## 5. Conclusions

High-temperature deoxidation experiments and inclusion-extraction experiments have been performed. The nucleation process of ZrO_2_ inclusions in Zr deoxidized steel was investigated by classical nucleation theory and first-principles calculation. The main conclusions are as follows.

When the Zr content was 100 ppm, SEM-EDS showed that the main inclusions in the steel were ZrO_2_. μXRD analysis confirmed the existence of ZrO_2_, and monoclinic and tetragonal ZrO_2_ were simultaneously detected, which may be because tetragonal ZrO_2_ transformed to monoclinic ZrO_2_ during the rapid cooling process. The average size of the ZrO_2_ inclusions was 0.56 μm. Through classical nucleation theory, the relationships between the solute element activities and the nucleation radius and nucleation rate of ZrO_2_ were obtained. The theoretical value of the nucleation rate was compared with the values in the literature, and the experimental value of *I* was approximately 40 orders of magnitude larger than the theoretical value.

The thermodynamic properties of macroscale ZrO_2_ were calculated by first principles, and the results were consistent with the experimental values. (ZrO_2_)*_n_* (*n* = 1–6) cluster models were constructed, and the thermodynamic properties of the geometrically optimized cluster structures and nanoscale ZrO_2_ particles were calculated, which verified the rationality of the existence of the pre-nucleation phase in terms of the thermodynamics. Based on two-step nucleation theory, the nucleation pathway of ZrO_2_ is proposed to be Zr_atom_ + O_atom_ → (ZrO_2_)*_n_* → (ZrO_2_)_2_ → core (ZrO_2_ particle)–shell ((ZrO_2_)_2_ cluster) nanoparticle → (ZrO_2_)_bulk_.

## Figures and Tables

**Table 1:** Chemical contents of the impurities in the pure iron sample (wt%).

Composition	C	Si	Mn	P	S	Cr	Al	Cu	Ni	Ti	N	Fe
Amount	0.016	0.0033	0.01	0.0053	0.0017	0.0107	0.003	0.0037	0.0038	0.001	0.002	Bal.

**Table 2:** Chemical contents of O, N, and Zr in the steel sample after Zr addition.

Zr Addition	Holding Time/s	[O]/ppm	[N]/ppm	[Zr]/ppm
		125	21	24
100 ppm	120	89	28	31
		106	34	22

**Table 3:** Data related to the calculation of the critical nucleation size and nucleation rate of oxide inclusions.

Inclusion	Θ (deg)	γ_SV_ (J/m^2^)	V_O_ (m^3^/mol)	logK_eq_
ZrO_2_	111 [27]	1.395 [27]	10.5× 10^−6^	−57,000/T + 21.8 [28]

**Table 4:** Interaction coefficients of O and Zr at 1873 K [26].

$e_{i}^{j}$	Zr	O
O	−23	−0.17
Zr	0.032	−4

**Table 5:** Structures, average bond lengths, sizes, and average binding energies of (ZrO_2_)*_n_* (*n* = 1–6) clusters.

Clusters	Bond Length(Zr-O) (nm)	$\mathrm{Size}\;(D_{max})\;\mathrm{nm}$	$E_{bin}$ eV
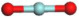 (ZrO_2_)_1_	0.189	0.38	−5.55
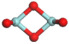 (ZrO_2_)_2_	0.192	0.48	−7.05
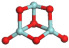 (ZrO_2_)_3_	0.204	0.60	−7.38
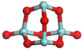 (ZrO_2_)_4_	0.20	0.66	−7.52
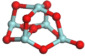 (ZrO_2_)_5_	0.206	0.70	−7.68
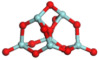 (ZrO_2_)_6_	0.203	0.93	−7.75

**Table 6:** LUMO–HUMO energy gaps of the (ZrO_2_)*_n_* (*n* = 1–6) clusters.

Clusters	(ZrO_2_)_1_	(ZrO_2_)_2_	(ZrO_2_)_3_	(ZrO_2_)_4_	(ZrO_2_)_5_	(ZrO_2_)_6_
LUMO	−0.15927	−0.12413	−0.15856	−0.15835	−0.11532	−0.13762
HOMO	−0.18301	−0.2138	−0.21179	−0.19151	−0.20248	−0.22429
LUMO-HUMO	0.023742	0.089665	0.05323	0.033158	0.087153	0.086679

## Data Availability

Part of the data presented in this study are available on request from the corresponding author. The data are not publicly available due to intellectual property.

## References

[1] Takamura J.I., Mizoguchi S. Roles of oxides in steel performance. Proceedings of The Sixth International Iron and Steel Congress, ISIJ International.

[2] Wang L., Yang S., Li J., Chen C., Li C., Li X. (2021). Study on the Capillary Interaction Between Particles on the Surface of High-Temperature Melts. Steel Res. Int..

[3] Li Y., Wang L., Li J., Yang S., Chen C., Li C., Li X. (2021). In-Situ Observation on the Agglomeration and Dispersion of Particles at the Interface of High-temperature Melts. ISIJ Int..

[4] Abraham S., Bodnar R., Raines J., Wang Y. (2018). Inclusion engineering and metallurgy of calcium treatment. J. Iron Steel Res. Int..

[5] Liu W., Yang S.F., Li J.S., Wang F., Yang H.B. (2019). Numerical model of inclusion separation from liquid metal with consideration of dissolution in slag. J. Iron Steel Res. Int..

[6] Peng B.W., Li F.J., Zheng S.B., Li H.G., Zhai Q.J. (2018). Inclusion control study in high aluminum steel. J. Iron Steel Res. Int..

[7] Zhao K.W., Zeng J.H., Wang X.H. (2009). Nonmetallic inclusion control of 350 km/h high speed rail steel. J. Iron Steel Res. Int..

[8] Wang L., Yang S., Li J., Wu T., Liu W., Xiong J. (2016). Improving Cleanliness of 95CrMo Drill Rod Steel by Slag Refining. Met. Mater. Trans. A.

[9] Yang Y., Zhan D., Lei H., Qiu G., Li Y., Jiang Z., Zhang H. (2019). In situ observation of acicular ferrite nucleation and growth at different cooling rate in Ti-Zr deoxidized steel. Metall. Mater. Trans. B.

[10] Chai F., Yang C.F., Su H., Zhang Y.Q., Xu Z., Yang Y.H. (2008). Effect of Zr addition to Ti-killed steel on inclusion formation and microstructural evolution in welding induced coarse-grained heat affected zone. Acta Metall. Sin. Engl. Lett..

[11] Guo A.M., Li S.R., Guo J., Li P.H., Ding Q.F., Wu K.M., He X.L. (2008). Effect of zirconium addition on the impact toughness of the heat affected zone in a high strength low alloy pipeline steel. Mater. Charact..

[12] Wu H.B., Hou M., Liang G.L., Tang D. (2012). Effect of zirconium on the low-temperature toughness of CGHAZ in F40 ship plates containing titanium. J. Univ. Sci. Technol. Beijing.

[13] Min J., Hu Z., Wang X., Pak J.J. (2013). Characterization of microstructure and non-metallic inclusions in Zr-Al deoxidized low carbon steel. ISIJ Int..

[14] Bhadeshia H.K.D.H., Honeycombe R.W.K. (2006). Steels Micro-Structure and Properties.

[15] He K., Baker T.N. (1996). Zr-contaning precipitates in a Ti-Nb micro-alloyed HSLA steel containing 0.016% Zr addition. Mater. Sci. Eng..

[16] Sarma D.S., Karasev A.V., Jonsson P.G. (2009). On the role of nonmetallic inclusions in the nucleation of acicular ferrite in steels. ISIJ Int..

[17] Guo Q.Y., Song B., Song M.M. (2019). Effects of Ti, Al and Zr deoxidation on morphology of sulfides in medium-sulfur non-quenched and tempered steel. Trans. Mater. Heat Treat..

[18] Li Y., Wan X.L., Cheng L., Wu K.M. (2014). First-principles calculation of the interaction of Mn with ZrO_2_ and its effect on the formation of ferrite in high-strength low-alloy steels. Scr. Mater..

[19] Wang G., Zhang L. (2012). Thermodynamics dependent on size in nucleation process of inclusions and calculation of critical nucleus radius in clean molten steel. Iron Steel.

[20] Wang G.C., Wang Q., Li S.L., Ai X.G., Fan C.G. (2014). Evidence of Multi-step Nucleation Leading to Various Crystallization Pathways from an Fe-O-Al Melt. Sci. Rep..

[21] Lei H., Xiao Y., Wang G., Zhang H., Jin W., Zhang L. (2020). Thermodynamic insight into the growth of nanoscale particle of Al-deoxidation in Fe–O–Al melt. Sci. Rep..

[22] Zhao D., Bao W., Li H., Zheng S., Chou K.-C. (2018). Cluster-assisted nucleation mechanism of titanium oxides in Fe-Ti supercooled alloys. J. Alloys Compd..

[23] Yang L., Zhang W., He L., Li H., Zheng S. (2019). Study on the growth and morphology evolution of titanium oxide clusters in molten iron with molecular dynamics simulation. RSC Adv..

[24] Suito H., Ohta H. (2006). Characteristics of Particle Size Distribution in Early Stage of Deoxidation. ISIJ Int..

[25] Wang L., Li J., Yang S., Chen C., Jin H., Li X. (2018). Nucleation and Ostwald Growth of Particles in Fe-O-Al-Ca Melt. Sci. Rep..

[26] Li Y., Wang L., Chen C., Li J., Li X. (2020). Effect of Mg Treatment on the Nucleation and Ostwald Growth of Inclusions in Fe-O-Al-Mg Melt. Materials.

[27] Humenik M., Kingery W.D. (1954). Metal-Ceramic Interactions: III, Surface Tension and Wettability of Metal-Ceramic Systems. J. Am. Ceram. Soc..

[28] Hino M., Ito K. (2010). Thermodynamic Data for Steelmaking.

[29] Ohta H., Suito H. (2006). Efects of Dissolved Oxygen and Size Distribution on Particle Coarsening of Deoxidation Product. ISIJ Int..

[30] Sakata K., Suito H. (1999). Dispersion of fine primary inclusions of MgO and ZrO_2_ in Fe-10 mass pct Ni alloy and the solidification structure. Met. Mater. Trans. B.

[31] Perdew J.P., Burke K., Ernzerhof M. (1997). Generalized Gradient Approximation Made Simple. Phys. Rev. Lett..

[32] Wang G., Xiao Y., Zhao C., Jing L., Shang D. (2017). Atomic Cluster Aggregates in Nucleation of Solid Alumina Inclusion in the Aluminum Deoxidation for Liquid Iron. Metall. Mater. Trans. B.

[33] Wang G., Xiao Y., Song Y., Zhou H., Tian Q., Li F. (2017). A density functional study on the aggregation of alumina clusters. Res. Chem. Intermed..

[34] Xiao Y., Lei H., Yang B., Wang G., Wang Q., Jin W. (2018). Nucleation and growth for magnesia inclusion in Fe-O-Mg melt. RSC Adv..

[35] Xiao Y., Lei H., Yang B., Zhao Y., Wang Q., Wang G. (2019). Thermodynamic insight into the growth of Calcia inclusions at the nanoscale: The case of Fe-O-Ca melt. RSC Adv..

[36] Aihara J.-I. (1999). Reduced HOMOLUMO Gap as an Index of Kinetic Stability for Polycyclic Aromatic Hydrocarbons. J. Phys. Chem. A.

[37] Chase M.W. (1998). NIST-JANAF Thermochemical Tables.

[38] Byun J.S., Shim J.H., Cho Y.W., Lee D.N. (2003). Non-metallic particle and intragranular nucleation of ferrite in Ti-killed C-Mn steel. Acta Mater..

[39] Phillpot S.R., Wolf D., Gleiter H. (1995). Molecular dynamics study of the synthesis and characterization of a fully dense, three dimensional nanocrystalline material. J. Appl. Phys..

